# Breaking Barriers: How Public Health Marketing Campaigns Are Reducing Stigma and Improving Early Psychosis Care

**DOI:** 10.1192/j.eurpsy.2026.11675

**Published:** 2026-07-31

**Authors:** G. Mcdermott, S. Chaudhry, V. Srihari

**Affiliations:** 1 Psychiatry, Yale Iniversity, New Haven; 2 Psychiatry, Tulane University, New Orleans, United States

## Abstract

**Introduction:**

*Comprehensive marketing campaigns can serve as powerful public health interventions to reduce the duration of untreated psychosis (DUP), a condition heavily burdened by stigma (Srihari et al, 2022). Red Rock Branding, an international communications firm contracted to develop psychosis awareness campaigns in three U.S. states, works with local clinicians and media specialists to craft targeted messaging. Central to these campaigns is the inclusion of people with lived experiences, whose voices humanize the condition and challenge stigmatized stereotypes that contribute to public health disparities (Hatzenbuehler et al, 2013). By centering personal narratives, Red Rock builds empathy, reduces fear, and dismantles barriers to treatment. This approach aims to shift public perceptions and encourage earlier helpseeking behavior, ultimately reducing DUP and improving long-term outcomes (Yee, et al, 2024).*

**Objectives:**

**Reframe marketing as an education process**

- Marketing *is* psychology. Understanding cognitive biases, emotional triggers, and decision-making helps psychologists communicate effectively without manipulation.
- Marketing isn’t about selling — it’s about helping more people get access to the care and resources they need.

**Methods:**

**The barriers you remove are as important as the services you provide.**

DUP is still an industry problem as many clients don’t follow through on seeking care because of, stigma, confusion about where to start, overwhelm with options.

**Clarity beats cleverness:** People in distress need clear, direct information.

**Social proof matters:** Testimonials, case studies, or even “you’re not alone” messaging reduce stigma and fear.

**Results:**

Our reputation has been established with in the public health sector over the last twelve years by successful campaigns with Yale, Tulane and Columbia universities.

We were published in a peer reviewed medical journal about the Mindmap campaign. With Mindmap, the Duration of Untreated Psychosis (DUP) was reduced from 10 months to 5 months. Details here

Our more recent work with Tulane University achieved a DUP from 15 months to 6 months. Video here and report here

We are in currently rolling our campaigns in Michigan and in discussions with Georgia on rolling out similar campaigns late 2025 and 2026

**Image 1:**
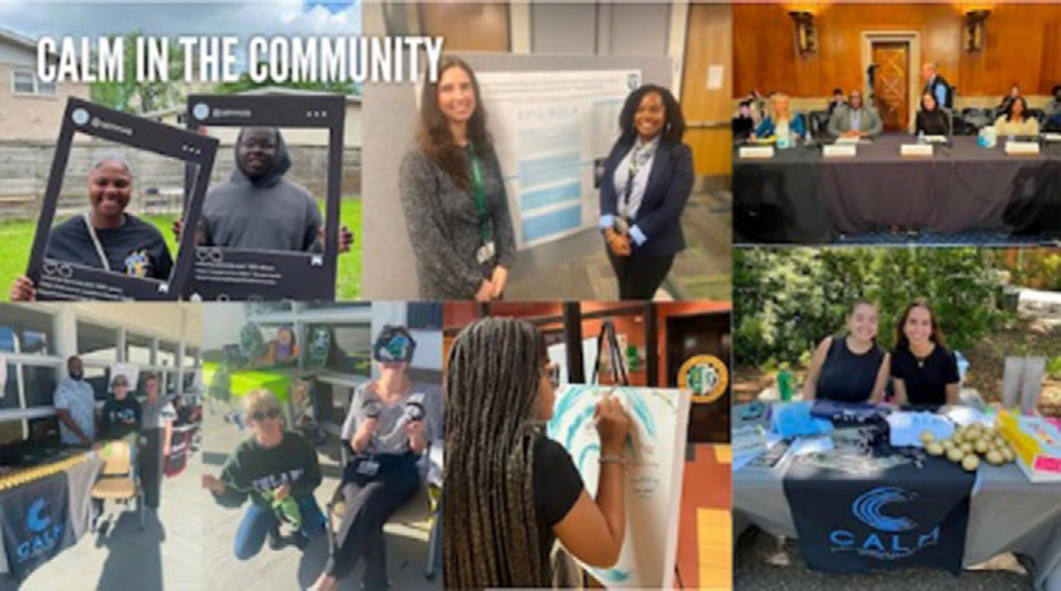
Image 1: Long description.

**Image 2:**
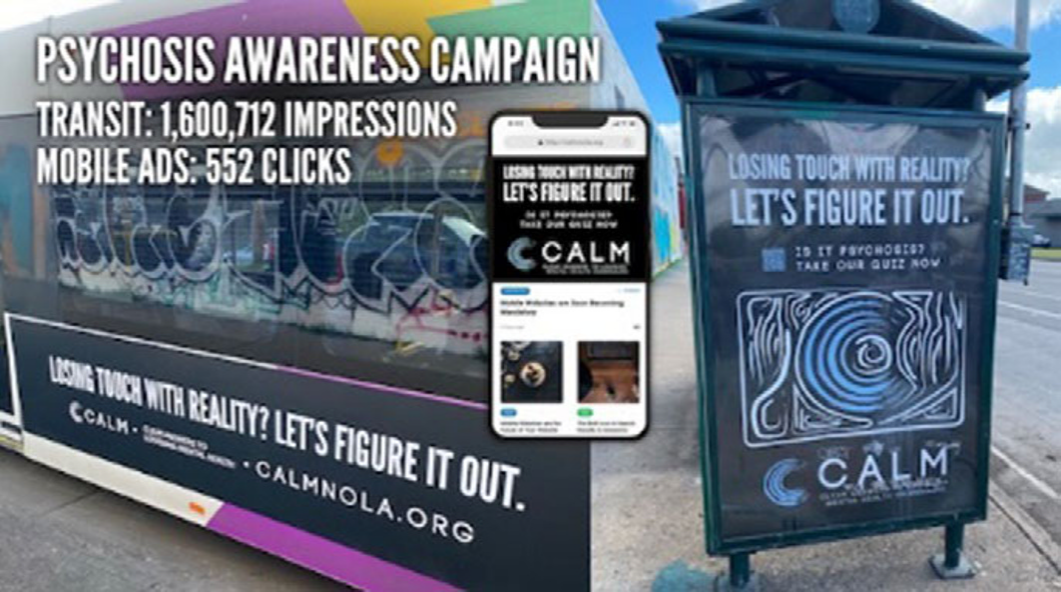

**Image 3:**
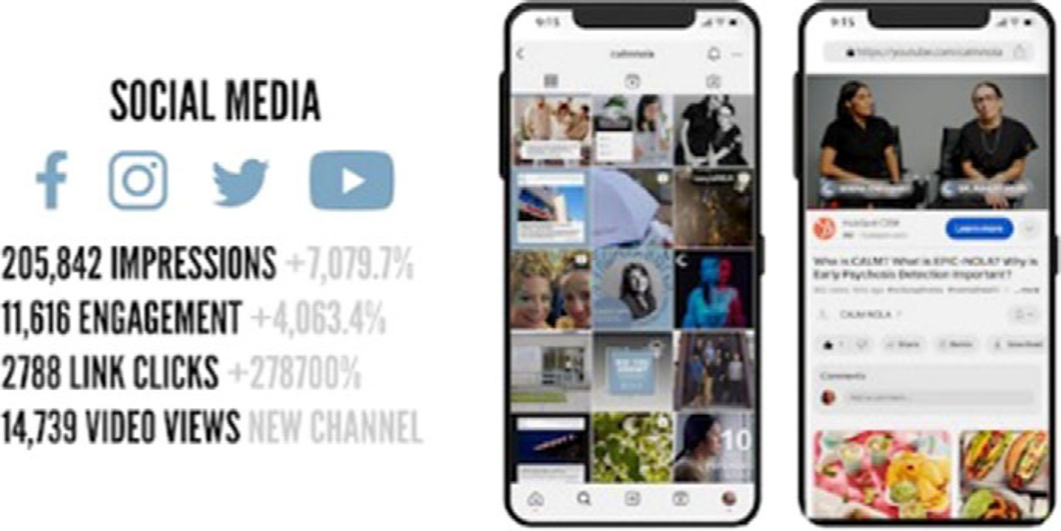
Image 3: Long description.

**Conclusions:**

*Red Rock’s campaign experience shows how empathy-driven interventions can reduce stigma, shorten DUP, and enhance both individual and public health outcomes.*

**Disclosure of Interest:**

None Declared

## Long descriptions

### Image 1: Long description

The collage features the text CALM IN THE COMMUNITY in white capital letters across the top-left.

* Top-left panel: Two individuals outdoors holding large black social media style photo frames.

* Top-center panel: Two researchers standing in front of a scientific poster titled E P I S N O L A.

* Top-right panel: Four people seated at a long table with microphones for a formal panel discussion in a wood-paneled room.

* Bottom-left panel: Three people standing behind a table with a blue C A L M logo banner outdoors.

* Bottom-center-left panel: Two people seated outdoors, one wearing a V R headset and holding controllers.

* Bottom-center-right panel: A person with long braids seen from behind, painting a blue circular design on a white canvas.

* Bottom-right panel: Two people smiling behind a table covered in merchandise and a blue C A L M banner in a park-like setting.

Navigate back to Image 1:.

### Image 3: Long description

The graphic is divided into three main sections from left to right.

1. Left Section: Statistical Summary

At the top, the text SOCIAL MEDIA is centered above four icons representing Facebook, Instagram, Twitter, and YouTube. Below the icons is a list of metrics with percentage increases in a lighter gray font:

* 205,842 IMPRESSIONS +7,079.7%

* 11,616 ENGAGEMENT +4,063.4%

* 2788 LINK CLICKS +278,700%

* 14,739 VIDEO VIEWS NEW CHANNEL

2. Middle Section: Instagram Interface

A smartphone displays an Instagram profile feed for the account calmnola. The grid shows twelve square posts featuring various photos of people, graphics with text, and outdoor scenes.

3. Right Section: YouTube Interface

A smartphone displays a YouTube video page. The video shows two women sitting in chairs during an interview. Below the video, the title reads Who is CALM? What is E P I C - N O L A? Why is Early Psychosis Detection Important? The channel name C A L M N O L A is visible below the title, followed by engagement buttons and a comment section.

Navigate back to Image 3:.

